# Their C-termini divide *Brassica rapa* FT-like proteins into FD-interacting and FD-independent proteins that have different effects on the floral transition

**DOI:** 10.3389/fpls.2022.1091563

**Published:** 2023-01-12

**Authors:** Areum Lee, Haemyeong Jung, Hyun Ji Park, Seung Hee Jo, Min Jung, Youn-Sung Kim, Hye Sun Cho

**Affiliations:** ^1^ Plant Systems Engineering Research Center, Korea Research Institute of Bioscience and Biotechnology (KRIBB), Daejeon, Republic of Korea; ^2^ Department of Biosystems and Bioengineering, KRIBB School of Biotechnology, University of Science and Technology (UST), Daejeon, Republic of Korea; ^3^ Department of Biotechnology, NongWoo Bio, Anseong, Republic of Korea; ^4^ Department of Biotechnology, Jenong S&T, Anseong, Republic of Korea

**Keywords:** Chinese cabbage, flowering time, flowering locus t (FT), FT–FD interaction, floral meristem identity genes

## Abstract

Members of the FLOWERING LOCUS T (FT)-like clade of phosphatidylethanolamine-binding proteins (PEBPs) induce flowering by associating with the basic leucine zipper (bZIP) transcription factor FD and forming regulatory complexes in angiosperm species. However, the molecular mechanism of the FT–FD heterocomplex in Chinese cabbage (*Brassica rapa* ssp. *pekinensis*) is unknown. In this study, we identified 12 *BrPEBP* genes and focused our functional analysis on four *BrFT-like* genes by overexpressing them individually in an *FT* loss-of-function mutant in *Arabidopsis thaliana*. We determined that *BrFT1* and *BrFT2* promote flowering by upregulating the expression of floral meristem identity genes, whereas *BrTSF* and *BrBFT*, although close in sequence to their Arabidopsis counterparts, had no clear effect on flowering in either long- or short-day photoperiods. We also simultaneously genetically inactivated *BrFT1* and *BrFT2* in Chinese cabbage using CRISPR/Cas9-mediated genome editing, which revealed that *BrFT1* and *BrFT2* may play key roles in inflorescence organogenesis as well as in the transition to flowering. We show that BrFT-like proteins, except for BrTSF, are functionally divided into FD interactors and non-interactors based on the presence of three specific amino acids in their C termini, as evidenced by the observed interconversion when these amino acids are mutated. Overall, this study reveals that although *BrFT-like* homologs are conserved, they may have evolved to exert functionally diverse functions in flowering *via* their potential to be associated with FD or independently from FD in *Brassica rapa*.

## Introduction

Shifting the timing of reproduction is a major objective of crop breeding efforts to develop new varieties that are better adapted to the changing climate conditions. As plants are grown in highly diverse environments with different temperatures and daylengths, these signals must be integrated by multiple networks to achieve successful reproduction (Bernier and Périlleux, 2005). Of these environmental cues, daylength and prolonged exposure to cold temperatures during winter (called vernalization) are the primary factors that control flowering time (Amasino and Michaels, 2010).

The genus *Brassica* is phenotypically diverse and comprises leafy vegetables, storage root vegetables, and oil crops; among them, Chinese cabbage (*Brassica rapa* L. ssp. *pekinensis*) is one of the most commercially important leafy vegetables in East Asia (Paterson et al., 2001; Cheng et al., 2016). Early flowering strongly decreases yield and quality as it restricts vegetative growth and leaf production; conversely, plants that never reach flowering will produce no seed. Timely flowering is therefore crucial for crop breeding, and understanding the molecular mechanism underlying flowering time is of great importance to prevent early flowering in Chinese cabbage.

Chinese cabbage (2n = 20, genome AA) is closely related to model plant Arabidopsis (*Arabidopsis thaliana*), as both are members of the *Brassicaceae* family. The genome sequence of the Chinese cabbage accession ‘Chiifu-401-42’ was released and uncovered 41,174 protein-coding genes (Wang et al., 2011). Although the regulatory pathways that control flowering time have been largely deciphered in Arabidopsis, much less is known about their counterparts in Chinese cabbage and how they integrate environmental cues to actuate flowering. Several quantitative trait loci (QTLs) have been identified for flowering time in Chinese cabbage and have uncovered the central flowering regulators *FLOWERING LOCUS C* (*FLC*) and *FLOWERING LOCUS T* (*FT*). Copies of *Bra.FLC.A02* (referred to as *BrFLC2*) and *Bra.FLC.A10* (referred to as *BraFLC1*) were associated with flowering time variation due to a 57-bp insertion/deletion (InDel) in the fourth exon and the fourth intron (*BraFLC2*) or aberrant splicing caused by a polymorphism in the 5′ splice site of the sixth intron (*BraFLC1*) leading to loss-of-function alleles (Yuan et al., 2009; Wu et al., 2012). A copy of *BrFT*, *Bra.FT.A07*, previously called *BrFT2* or *Bra.FT.b*, was the causal gene for a flowering QTL and harbors a transposon insertion in the second intron of the gene (Zhang et al., 2015; Wei et al., 2022). Another *BrFT* copy, *Bra.FT.a* (referred to as *BrFT1*), is also crucial for the initiation of the floral transition (Del Olmo et al., 2019). Notably, a systematic characterization of gene function for each of the multiple copies of paralogous flowering genes following the whole-genome triplication in Chinese cabbage is lacking.

*FT* is a central integrator of environmental and endogenous signals that modulate flowering. FT is a mobile protein that is translated in leaves and is transmitted to the shoot apical meristem, meeting the criteria of florigen, the long-distance signal that induces flowering (Corbesier et al., 2007). Vernalization allows production of a systemic signal FT by relieving transcriptional repression of FLC, being a result promoting transition to flowering (Searle et al., 2006). Molecular and genetic studies have revealed that FT directly interacts with the basic leucine zipper (bZIP) transcription factor FD at the shoot apex *via* an initial interaction with 14-3-3 proteins that act as intracellular FT receptors in the cytoplasm. The FT–14-3-3 complex then forms a ternary complex with FD in the nucleus (Wigge et al., 2005; Taoka et al., 2011). This complex can activate the transcription of floral identity genes such as *APETALA1* (*AP1*) and *SQUAMOSA PROMOTER BINDING PROTEIN LIKE*s (*SPLs*) in the shoot apex (Schmid et al., 2003; Abe et al., 2005; Wigge et al., 2005). FT-like proteins are 20 kDa in size and show homology to phosphatidylethanolamine-binding proteins (PEBPs). FT-like members include FT, TERMINAL FLOWER 1 (TFL1), TWIN SISTER OF FT (TSF), MOTHER OF FT AND TFL (MFT), and BROTHER OF FT AND TFL1 (BFT), which have all been shown to behave as activators or repressors of flowering in Arabidopsis (Karlgren et al., 2011). However, not much is known about *FT-like* genes in Chinese cabbage.

Here, we identified four *FT* homologs (*BrFT1*, *BrFT2*, *BrTSF*, and *BrBFT*, collectively called *BrFT-like* genes) with the highest sequence identity to Arabidopsis FT among the 12 PEBP proteins encoded by the Chinese cabbage genome. The individual ectopic expression of these genes in the Arabidopsis loss of function FT allele (*ft-10*) revealed the functional divergence between the *BrFT-like* genes. Indeed, *BrFT1* and *BrFT2* accelerated flowering, whereas *BrTSF* and *BrBFT* failed to rescue the late flowering phenotype of the *ft-10* mutant as determined by expression levels of floral meristem identity genes. In a complementary approach, we obtained simultaneously loss-of-function mutants for *BrFT1* and *BrFT2* in Chinese cabbage through clustered regularly interspaced short palindromic repeat (CRISPR)/CRISPR-associated nuclease 9 (Cas9)-mediated gene editing. The characterization of these Chinese cabbage mutants supported the idea that *BrFT1* and *BrFT2* play a key role in flowering time regulation. Moreover, we found that BrFDs differentially interact with BrFT-like proteins based on the presence of a conserved three–amino acid motif in the C termini of BrFTs. Our results suggest that although PEBP homologs are conserved in *B. rapa*, they contribute to the observed diversity for flowering time regulation.

## Results

### Molecular characterization of *BrFT-like* genes in Chinese cabbage

To identify PEBP family members in Chinese cabbage (*BrPEBP*), we analyzed our previous RNA-seq dataset (Jung et al., 2021) with the amino acid sequence of Arabidopsis FT (AtFT, encoded by At1g65480) and identified 12 candidate genes (**Table S1**). In agreement with the previous study in the *B. rapa* variety ‘yellow sarson R-o-18’ (Del Olmo et al., 2019), the protein encoded by Bra022475 (referred to as BraA.FT.a in this study) showed the highest identity to AtFT with 86%, followed by the proteins encoded by Bra004117 (BraA.FT.b) and Bra015710 (BraA.TSF) with 82%, and that encoded by Bra010052 (BraA06g025510) with 60%. Other PEBP-like proteins shared less than 60% in identity with AtFT and were not considered further.

**Figure 1:**
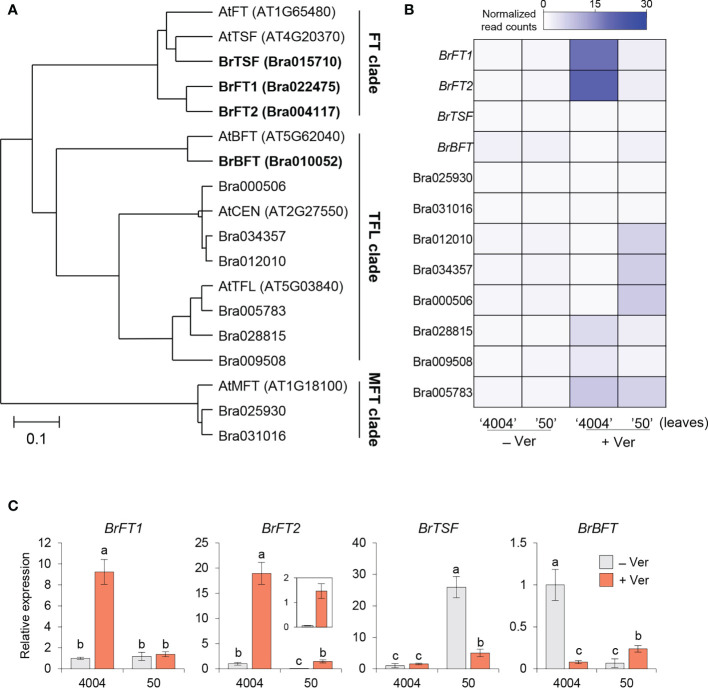
Identification of FT-like proteins from PEBP candidates in Chinese cabbage (*Brassica rapa* subsp. *pekinensis*). **(A)** Phylogenetic tree of PEBP members from Arabidopsis and Chinese cabbage. The tree was constructed using Bayesian evolutionary analysis in BEAST 2.5 (version 2.7.1). The scale bar represents the 95% highest posterior density (HPD) interval for the age of each node in the tree. PEBPs cluster into three clades. **(B)** Heatmap representation of the expression levels of *Bra PEBP* candidates based on RNA-seq analysis in Chinese cabbage. Columns represent the two Chinese cabbage inbred lines (4004; early bolting, 50; late bolting) exposed (+, for 35 days) or not (–) to vernalization treatment. RNA-seq was performed using the leaf samples of Chinese cabbage with or without vernalization. Normalized read counts were calculated from the mean of three biological replicates. **(C)** RT-qPCR analysis of *BrFT-like* gene expression in the two inbred lines 4004 and 50. *BrActin 2* (*BrACT2*) was used for normalization. For each gene, the expression level in non-vernalized 4004 was set to 1. Data are means ± SE of three biological replicates. Different lowercase letters represent significant differences, as determined by one-way ANOVA followed by Tukey’s *post-hoc* test (*P* < 0.05).

To characterize the structural and functional divergence of the BrPEBP candidates, we performed a phylogenetic tree analysis using the Bayesian evolutional analysis software called BEAST 2.5 (Bouckaert et al., 2019). The phylogenetic analysis also included five Arabidopsis PEBP family members, TSF, BFT, CENTRORADIALIS (CEN), and MFT for putative functional assignment (Jin et al., 2021). The BrPEBP candidates were divided into three groups named FT-, TFL-, and MFT- clade with three Bra022475, Bra004117 and Bra015710 belonging to FT clade. In detail, the proteins encoded by Bra022475 and Bra004117 were the closest to AtFT, prompting us to rename their encoding genes *BrFT1* and *BrFT2*, respectively. The protein encoded by Bra015710 was close to AtTSF than AtFT, while the protein encoded by Bra010052 was close to AtBFT in TFL clade; the corresponding genes were thus renamed *BrTSF* and *BrBFT*, respectively. The other Chinese cabbage proteins in the tree were closer to AtCEN, AtTFL, or AtMFT (**Figure 1A**). We thus focused on *BrFT1*, *BrFT2*, *BrTSF*, and *BrBFT* for further analysis as potential *AtFT*-like genes.

To characterize the function of the *BrPEBP* genes above, we examined their transcript levels using our previous RNA-seq dataset derived from the early-bolting inbred line ‘4004’ and the late-bolting inbred line ‘50’ grown under normal conditions (continuous condition at 23°C, 16 h light/8 h dark) or exposed to vernalization (at 4°C for 35 days at the same light conditions) (Jung et al., 2021). We determined that *BrFT1* and *BrFT2* are relatively highly expressed in inbred line 4004, but not in inbred line 50 in response to vernalization in accordance with the flowering phenotypes between the two inbred lines. From this result, we suggest a promoting role in flowering for these two genes (**Figure 1B**, lane 3). *BrTSF*, *BrBFT*, and other *PEBP*-*like* genes were rarely expressed under either normal or vernalization conditions (**Figure 1B**). To confirm these expression levels, we performed reverse transcription quantitative PCR (RT-qPCR) analysis of the four *BrFT*-like genes in 4004 and 50 plants exposed to vernalization or maintained at 22°C. The expression level of *BrFT1* rose about 9-fold in the 4004, but not in 50 upon vernalization. Although *BrFT2* was expressed 13-fold more highly in 4004 than in 50, *BrFT2* expression levels increased in both lines in response to vernalization, with a 19-fold and 29-fold increase in 4004 and 50, respectively. Notably, *BrTSF* expression did not appear to respond to vernalization in the 4004 line and decreased 5-fold in line 50. *BrBFT* showed opposite responses to vernalization in its transcript levels in line 4004, experiencing a 14-fold drop following vernalization compared to normal conditions, whereas its expression level increased about 3.5-fold after vernalization treatment in line 50 (**Figure 1C**). We conclude that *BrFT1* and *BrFT2* may differ from *BrTSF* and *BrBFT* in terms of their response to vernalization, raising the possibility that these two groups of genes may differently regulate flowering time.

### BrFT1 and BrFT2 are floral activators, but BrTSF and BrBFT are undefined function proteins in Arabidopsis

To assess the roles of *BrFT-like* genes in the regulation of flowering time, we individually overexpressed the full-length genomic sequence from *BrFT-like* genes (*gBrFT1*, *gBrFT2*, *gBrTSF*, and *gBrBFT*) in the Arabidopsis *ft-10* mutant (**Figure S1A**) under the control of the cauliflower mosaic virus (CaMV) 35S promoter (**Figure S1B**). Overexpressing *BrFT1* or *BrFT2* in the *ft-10* mutant background accelerated flowering compared to the vector control, or the overexpression of *BrTSF* or *BrBFT*, in both the T_1_ and T_2_ generations (**Figures S2A, B**). We confirmed the overexpression of individual *BrFT-like* genes in T_2_ transgenic lines by RT-qPCR analysis (**Figure S2C**). We then selected homozygous T_3_ transgenic lines by selecting seedlings on half-strength Murashige and Skoog (MS) medium containing hygromycin and confirmed the presence of the transgene by PCR analysis (**Figure S2D**).

**Figure 2:**
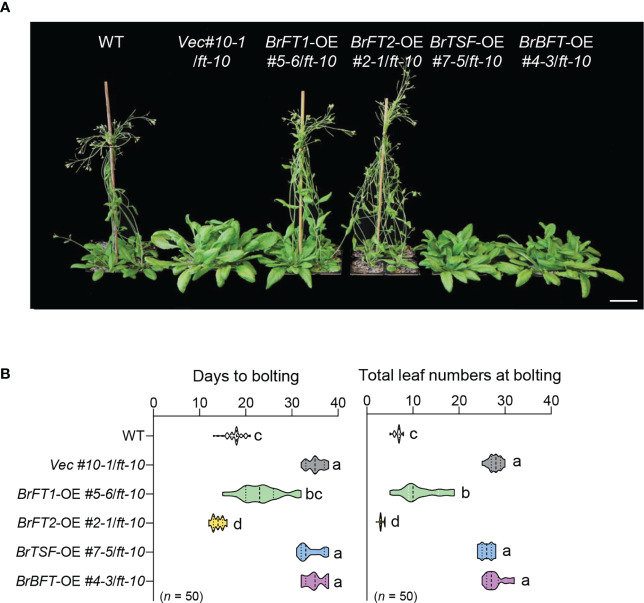
Ectopic expression of *BrFT1* and *BrFT2* causes flowering in the Arabidopsis *ft-10* mutant. **(A)** The ectopic expression lines *BrFT1-OE* #5-6/*ft-10*, *BrFT2-OE* #2-1/*ft-10*, *BrTSF-OE* #7-5/*ft-10*, or *BrBFT-OE* #4-3/*ft-10* and the empty vector control (*Vec* #10-1/*ft-10*) were compared with the wild type (WT, ecotype Col-0). Plants were grown at 23°C under LD conditions for 30 days. Scale bar, 5 cm. **(B)** Distribution of flowering phenotypes (as days to bolting [left] and number of rosette leaves [right]) in T_3_ lines, shown as violin plots. Five biological replicates were performed for analysis (*n* of each replicate ≥ 10). Different lowercase letters represent significant differences, as determined by one-way ANOVA followed by Tukey’s *post-hoc* test (*P* < 0.05).

We selected one homozygous transgenic line per *BrFT-like* gene: *BrFT1-OE* #5-6/*ft-10* for *BrFT1*, *BrFT2-OE* #2-1/*ft-10* for *BrFT2*, *BrTSF-OE* #7-5/*ft-10* for *BrTSF*, and *BrBFT-OE* #4-3/*ft-10* for *BrBFT*, with *Vec* #10-1/*ft-10* as empty vector control. Homozygous *BrFT1-OE* #5-6/*ft-10* or *BrFT2-OE* #2-1/*ft-10* transgenic plants flowered much earlier than the *Vec* #10-1/*ft-10* control when grown under long-day conditions (**Figure 2A**), flowering after 22 days or 15 ± 7 days, respectively, and produced far fewer leaves than the *Vec* #10-1/*ft-10* control with a number coming close to that of the wild-type Col-0 accession (background of *ft-10*) (**Figure 2B**). In sharp contrast, homozygous lines overexpressing *BrTSF* or *BrBFT* (lines *BrTSF-OE* #7-5/*ft-10* and *BrBFT-OE* #4-3/*ft-10*) flowered at the same time as *Vec* #10-1/*ft-10*, at 35 ± 3 days after sowing, and produced the same number of leaves as the empty vector control.

To corroborate the relative expression levels of *BrFT-like* genes in flowering time, we performed RT-qPCR analysis and confirmed that each *BrFT-like* gene is overexpressed in its corresponding transgenic line in the *ft-10* background (**Figure S2E**). These results suggest that *BrFT1* and *BrFT2* are important in flowering time regulation and function as floral activators. We obtained the same results with the independent T_3_ transgenic lines *BrFT1-OE* #10-4/*ft-10* and *BrFT2-OE* #8-3/*ft-10*, which both accelerated flowering to a similar extent as *BrFT1-OE* #5-6/*ft-10* and *BrFT2-OE* #2-1/*ft-10* (**Figure S3**). By contrast, *BrTSF* and *BrBFT* did not appear to contribute to flowering time regulation, at least when overexpressed in Arabidopsis.

### Ectopic expression of *BrFT1* and *BrFT2* activates floral homeotic genes

**Figure 3:**
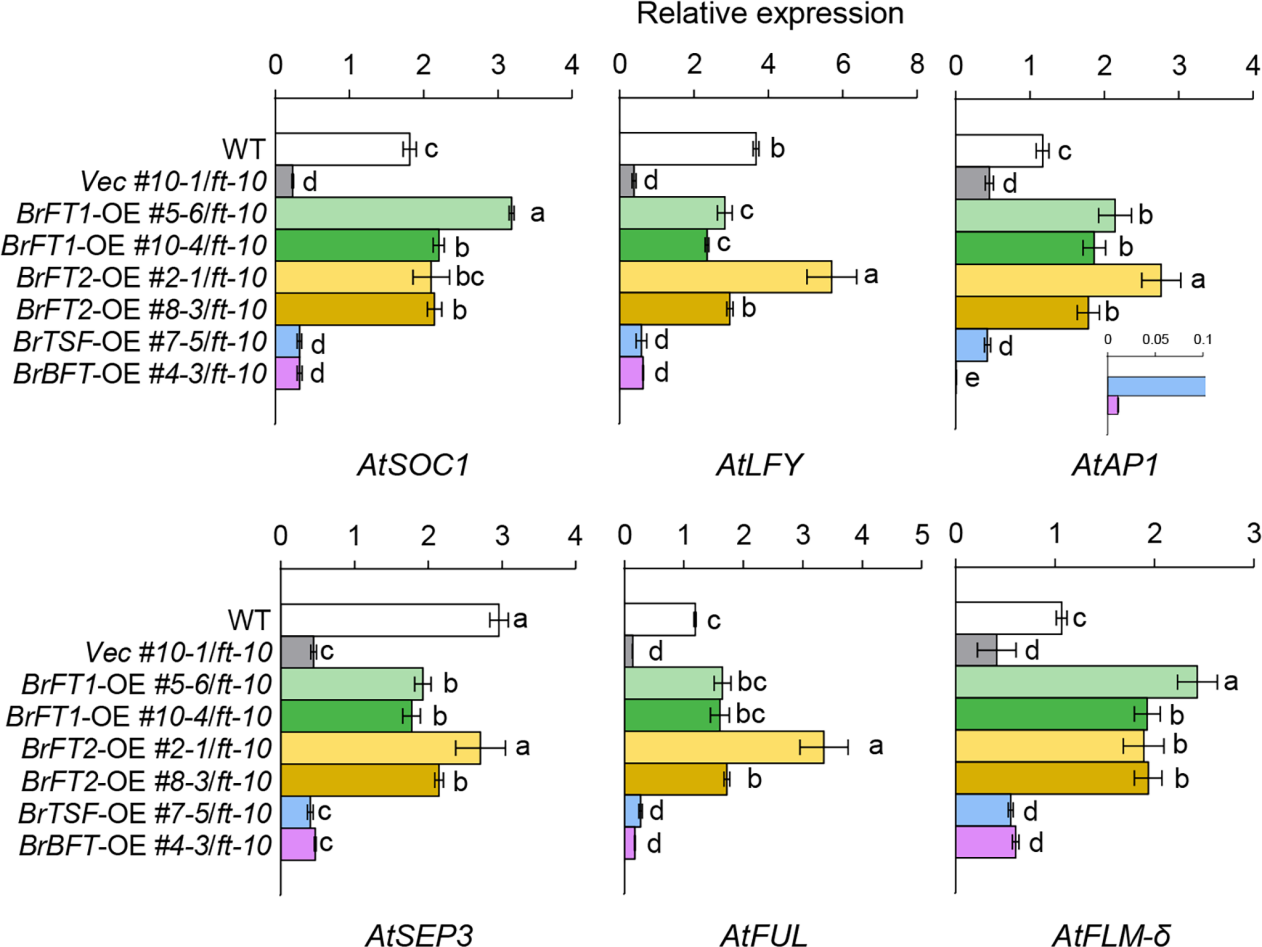
RT-qPCR analysis of flowering time genes in lines overexpressing *BrFT-lik*e genes in Arabidopsis. This analysis was done on the leaf samples from the same location grown for 2 weeks. Gene expression levels were normalized to *AtACT2* as a reference. Data are means ± SE of three biological replicates. Different lowercase letters represent significant differences, as determined by one-way ANOVA followed by Tukey’s *post-hoc* test (*P* < 0.05).

To determine whether *BrFT-like* genes induce a subset of genes whose expression is known to be regulated by the FT–FD complex, we analyzed the expression levels of floral meristem identity genes in Arabidopsis transgenic lines overexpressing *BrFT1* or *BrFT2* by RT-qPCR analysis. Indeed, the transcript level of *AtSOC1* (*SUPPRESSOR OF OVEREXPRESSION OF CO 1*), whose expression can be directly induced by the FT–FD complex (Lee and Lee, 2010), significantly increased in *BrFT1* and *BrFT2* transgenic plants compared to the vector control line and even higher than the Col-0 control. The expression of *LEAFY* (*LFY*), the master regulator of floral fate and another target gene of the FT–FD complex (Zhu et al., 2020), was also highly induced by the overexpression of *BrFT1* or *BrFT2* in *ft-10* and reached levels close to that of Col-0. In addition, the floral homeotic genes *AP1*, *FRUITFUL* (*FUL*), and *SEPALLATA 3* (*SEP3*), whose expression is activated in response to the FT–FD complex (Teper-Bamnolker and Samach, 2005; Wigge et al., 2005), increased relative to the vector control line and reached levels comparable to Col-0 when *BrFT1* or *BrFT2* was overexpressed (**Figure 3**; green and yellow color series graphs).

The splice *FLOWERING LOCUS M* (*FLM*) isoform *AtFLM-δ* encodes a floral promoter in Arabidopsis (Capovilla et al., 2017); overexpressing *BrFT1* or *BrFT2* in *ft-10* increased *AtFLM-δ* levels 2- to 3-fold relative to Col-0 and the empty vector control line (**Figure 3**; green and yellow color series graphs). By contrast, overexpression of *BrTSF* or *BrBFT* did not affect the expression levels of floral homeotic identity genes or *FLM-δ* in the *ft-10* mutant background, with the exception of *AtAP1* (**Figure 3**; sky blue and pink color graphs). Interestingly, the *BrBFT* transgenic line showed an extremely low expression of *AtAP1* compared to all other transgenic lines and Col-0 WT. We conclude that the overexpression of *BrFT1* or *BrFT2* can induce the expression of a subset of floral meristem identity genes, as does *AtFT*.

### A CRISPR/Cas9-mediated loss-of-function mutation in both *BrFT1* and *BrFT2* impairs floral organogenesis and flowering time in Chinese cabbage

**Figure 4:**
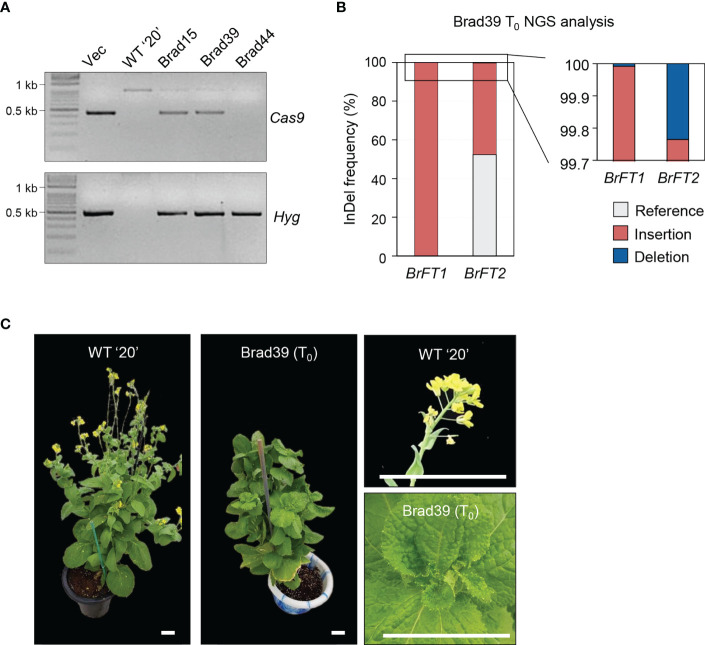
Generation of CRISPR/Cas9-mediated gene-edited plants for both *BrFT1* and *BrFT2* in Chinese cabbage inbred line ‘20’ and associated flowering phenotypes. **(A)** Screening of T_0_ plants. PCR was performed to identify *BrFT1/2*-edited Chinese cabbage plants. **(B)** Analysis of Brad39 (T_0_) plant by whole-genome sequencing. Percentage represents the proportion of reference and InDel (insertion/deletion) alleles at the target loci. Sequence alignments between the WT and mutants are shown in **Figure S4B**. **(C)** Bolting phenotype of WT (‘20’ inbred line) and a genome-edited Brad39 (T_0_) plant. The photographs to the right show the flower buds (20) or the main stem still producing leaves (Brad39) at the same age. Scale bars, 5 cm.

The similar phenotypes upon overexpression of *BrFT1* or *BrFT2* in the *ft-10* mutant raised the possibility that they might regulate flowering time redundantly in Chinese cabbage. To test this hypothesis, we used simultaneous CRISPR/Cas9-mediated mutagenesis of *BrFT1* (Bra022475) and *BrFT2* (Bra004117) genes using the Chinese cabbage inbred line ‘20’ as in our previous study (Jung et al., 2021). We designed one single-guide RNA (sgRNA) targeting the first exon of *BrFT1* and *BrFT2* to edit both genes simultaneously (**Figure S4A**). We then transformed Chinese cabbage hypocotyls and regenerated whole plants, as described in Materials and Methods. We obtained several T_0_ plants that we genotyped for the presence of mutations at the target sites by genotyping PCR and whole-genome sequencing (**Figure 4A**). We selected line Brad39, which mutation was of > 99% for *BrFT1* and > 50% for *BrFT2* with insertion/deletion accounting for the largest proportion of mutations using next-generation sequencing (NGS) (**Figure 4B**, **Figure S4B**). We then grew the genome-edited ‘Brad39’ T_0_ plant under the same growth conditions as its isogenic wild-type parent 20 to obtain T_1_ seeds, but Brad39 did not bolt even after 3 months of growth (**Figure 4C**). Even after 6 months in LD conditions, Brad39 failed floral organogenesis thus never produced T_1_ seeds, as the switch to the reproductive stage never took place (**Figure S5**). We thus concluded that of the four *BrFT-like* homologs in Chinese cabbage, *BrFT1* and *BrFT2* may be redundant positive regulators of flowering whose simultaneous loss of function impairs floral organogenesis.

### BrFT-like proteins interact with FDs *via* highly selective amino acid residues in their C termini

Since FT regulates the transcription of a subset of downstream genes by interacting with FD (Abe et al., 2005), we further investigated whether BrFT-like proteins interact with BrFD protein. Previous reports have indicated that Arabidopsis FT harbors four segments (segments A to D) in its C terminus and that FT interacts with FD through the formation of an external loop formation of 14 amino acids from segment B, while the L/IYN motif in segment C is crucial for FT activity (Ahn et al., 2006; Wang et al., 2017). We first aligned BrFT-like proteins, AtFT, AtTSF, AtBFT, and the rice floral integrator Heading date 3a (OsHd3a) to explore the extent of sequence conservation (**Figure S6**). In segment B, the 14–amino acid sequence from BrBFT was more consistent with that of AtBFT than AtFT; moreover, in segment C, BrBFT did not have the same motif as other BrFT-like proteins and BrTSF showed the motif NYN, thus harboring a mismatched sequence. We hypothesized that the differences in sequence between BrTSF/BrBFT and BrFT1/BrFT2 might determine their interaction potential with FD.

**Figure 5:**
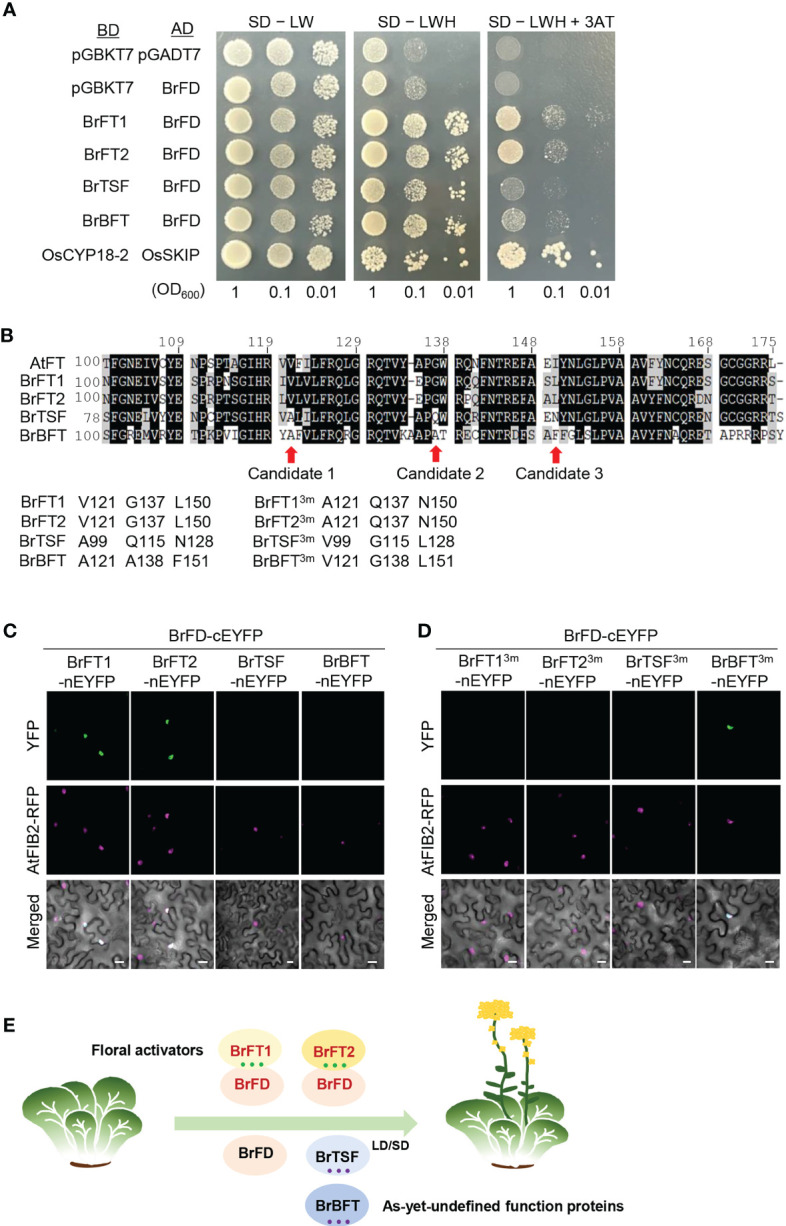
Protein–protein interactions between BrFT-like proteins and BrFD. **(A)** Yeast two-hybrid assays testing the interaction between BrFT-like proteins and BrFD. BrFT-like proteins in the pGBKT7 vector and BrFD in pGADT7 vector were used as bait and prey constructs, respectively. The empty pGBKT7 was used as negative control, and OsCYP18-2 and OsSKIP were used as the positive control. AH109 yeast cells were grown on synthetic defined (SD) medium lacking tryptophan and leucine (SD –LT); SD lacking tryptophan, leucine, and histidine (SD –LTH); and SD medium lacking tryptophan, leucine, and histidine and containing 3 mM 3-amino-1,2,4-triazole (3AT) (Sd –LTH + 3AT). **(B)** Sequence alignment of the C-terminal domain of AtFT and BrFT-like proteins. Val-121, Gly-137, and Leu-150 were selected as key amino acids that are distinct between AtFT, BrFT1/2, and BrTSF/BrBFT. **(C)** BiFC assay testing the interactions between BrFT-like proteins and BrFD. Constructs encoding BrFT-like proteins fused to the N-terminal half of eYFP (nEYFP) were co-expressed with a construct encoding BrFD fused to the C-terminal half of eYFP (cEYFP) in *N. benthamiana* leaves. Scale bars, 20 μM. **(D)** BiFC assay with constructs encoding BrFT-like proteins harboring three–amino acid substitutions (BrFT^3ms^) fused to nEYFP and co-expressed with a construct encoding BrFD fused to cEYFP in *N. benthamiana* leaves. Scale bars, 20 μM. **(E)** A proposed model for the functional diversification of BrFT-like proteins *via* their interaction with BrFD. BrFT1 and BrFT2 interact with BrFD to facilitate flowering. By contrast, BrTSF and BrBFT do not interact with BrFD and do not activate flowering.

To determine whether BrFT-like proteins interact with BrFD, we performed a yeast two-hybrid (Y2H) assay. To this end, we individually cloned the full-length coding sequences of *BrFT*-like genes into pGBKT7 (BD vector), while the full-length coding sequence of *BrFD* was cloned into pGADT7 (AD vector). We introduced the appropriate pairs of constructs into yeast cells and tested protein–protein interaction, which revealed that BrFT1 and BrFT2 can interact with BrFD, whereas BrTSF and BrBFT did not (**Figure 5A**). Next, to delineate the exact differences between BrFT-like proteins that dictate their interaction with BrFD, we took a closer look at the protein alignment of BrFT-likes with AtFT and noticed that three amino acids (aa), Val-121, Gly-137, and Leu-150, are distinct between positive regulators of flowering (BrFT1 and BrFT2) and undefined function proteins (BrTSF and BrBFT) (**Figure 5B**). To examine whether the substitution of these three aa in BrFT-like proteins might change their interaction with FD, we performed bimolecular fluorescence complementation (BiFC) assays by transiently infiltrating *Nicotiana benthamiana* leaves with constructs encoding BrFT-like proteins fused to the N-terminal half of enhanced yellow fluorescence protein (BrFT-like-nEYFP) and BrFD fused to the C-terminal half of EYFP (cEYFP-BrFD). We observed green fluorescence in the nucleus when *BrFT1* and *BrFT2* constructs were co-expressed with the *BrFD* and *AtFD* constructs, but not when the FD constructs were co-expressed with either *BrTSF* or *BrBFT*, in line with the Y2H result (**Figure 5C** and **S7A**). Next, we reciprocally changed the three aa of BrFT1, BrFT2, BrTSF, and BrBFT and repeated the BiFC assay (**Figure 5B**: each 3m). Surprisingly, BrFT1^3m^ and BrFT2^3m^ lost their ability to bind to the two FDs, while BrBFT^3m^ gained binding activity toward BrFD and AtFD (**Figure 5D**, **S7B**). Changing these three aa in BrTSF failed to confer the ability to interact with BrFD or AtFD for an unknown reason.

### BrTSF is incapable of inducing flowering in the Arabidopsis *ft-10* mutant

Although *BrTSF* overexpression did not rescue the late flowering of *ft-10* and its encoded protein failed to interact with FD, we grew transgenic lines overexpressing *BrTSF* under short-day (SD) conditions, since Arabidopsis *TSF* promotes flowering under this condition (Yamaguchi et al., 2005). When grown in SDs, most *BrFT1-OE #5-6*/*ft-10* and *BrFT2-OE #2-1*/*ft-10* plants reached the flowering stage 5 weeks after germination, thus 2 to 3 weeks faster than Col-0. However, the overexpression line *BrTSF-OE* #7-5/*ft-10* showed no flowering after 8 weeks, like *Vec* #10-1/*ft-10* plants. Similarly, the other overexpression line *BrBFT-OE* #4-3/*ft-10* failed to flower after 9 weeks in SDs (**Figures S8A, B**). In fact, none of the plants from the *Vec* #10-1/*ft-10*, *BrTSF-OE* #7-5/*ft-10*, or *BrBFT-OE* #4-3/*ft-10* transgenic lines reached the flowering stage in SDs after close to 13 weeks (90 days), in contrast to Col-0 and lines overexpressing *BrFT1* and *BrFT2* (**Figures S8C, D**). We conclude that *BrTSF* is incompetent to induce flowering in Arabidopsis *ft-10* mutant under both LD and SD conditions.

## Discussion

The genomes of most important crop plants have evolved through extensive gene duplications or by whole-genome polyploidization, resulting in diversification of duplicated genes over time, particularly for flowering time (Masterson, 1994). The additive or dosage-dependent effects of key regulatory genes present in multiple copies in *Brassica* species and controlling flowering time have been reported (Schranz et al., 2002; Chen et al., 2021; Jung et al., 2021). However, how these genes are retained over the course of evolution and what their underlying mechanisms are in the control of flowering time are largely unknown in *Brassica rapa*. Our findings support the notion that *BrFT-like* genes contribute to flowering time variation that relies on their interaction with FD *via* three critical amino acids in the C termini of their encoded proteins (**Figure 5E**).

In a recent study, 13 *B. rapa FT-like* candidate genes were identified using Arabidopsis *FT* as a query in the *B. rapa* Chiifu-401 v3.0 genome (Del Olmo et al., 2019). Most of the *BrPEBP* candidate genes identified here were consistent with this earlier study, with the exception of the Arabidopsis *CEN/ATC* homolog BraA04g019800, whose expression we did not detect in our RNA-seq data (**Figure 1A**). We therefore defined 12 *B. rapa FT* homologs. Nevertheless, our molecular characterization of four *BrFT-like* genes contradicted the characterization of *BrFT2* (referred to as *BraA.FT.b* in the previous study). Del Olmo et al. failed to amplify genomic region of *BrFT2* and did not detect expression of this gene in *B. rapa* leaves, reaching the conclusion that *BrFT2* was a nonfunctional gene. By contrast, our results revealed that *BrFT2* responded strongly to vernalization at the transcriptional level (**Figures 1B, C**). In addition, we successfully cloned the *BrFT2* genomic region from *B. rapa* L. *pekinensis* (**Figure S1**). To date, there have been no reports on *TSF* or *BFT* functions in *Brassica* species. However, *FT* is typically represented by a multigene family in various crops. The soybean genome possesses at least 10 *FT* genes, a subset of which promote flowering (*GmFT2a/2b*, *GmFT3a/3b*, and *GmFT5a/5b*), while *GmFT1a/1b*, *GmFT4*, and *GmFT6* repress flowering (Lee et al., 2021). Sugar beet (*Beta vulgaris*) has two *FT* genes (*BvFT1* and *BvFT2*) with opposite functions in flowering as well as different expression patterns (Pin et al., 2010). Onion (*Allium cepa*) also has six *FT* homologs, with *AcFT1* and *AcFT2* acting as floral promotors, whereas *AcFT4* delays flowering (Lee et al., 2013). Thus, the relative dosage of *FT-like* genes and their transcripts may be important for optimizing flowering time during growing seasons in various plant species.

We showed here that BrFT1, BrFT2, BrTSF, and BrBFT from PEBP/FT-like proteins were closer in sequence to AtFT among all 12 BrPEBP-like proteins, which prompted us to focus on their characterization in flowering time. We individually overexpressed *BrFT1* or *BrFT2* in the Arabidopsis late flowering mutant *ft-10* and observed the near complete rescue of its delayed flowering. By contrast, the overexpression of *BrTSF* or *BrBFT* had no effect on the flowering time of *ft-10* (**Figure 2**). These results are consistent with the previously identified QTLs for *BrFTb* (Zhang et al., 2015) and ethyl methanesulfonate (EMS)–mediated mutagenesis of *Bra.A.FTa* (Del Olmo et al., 2019), although no results have been presented about their functional equivalency and redundancy. In Arabidopsis, *TSF* is highly homologous to *FT*, and overexpressing *TSF* leads to an early flowering phenotype, as does overexpressing *FT* (Kobayashi et al., 1999; Yamaguchi et al., 2005), placing *TSF* as an essential player in the regulation of flowering time in Arabidopsis. However, our results indicated that overexpressing *BrTSF* had no effect on flowering time, despite its high sequence identity to FTs. We also tested whether *BrTSF* functioned specifically under SD conditions as in previous studies conducted in Arabidopsis (Yamaguchi et al., 2005), but again we did not observe an effect on flowering time in *BrTSF* overexpressing plants (**Figure S8**). BrBFT belonged to the same clade as BrTSF, which was distinct from FT Clade proteins above, and neither accelerated flowering time when overexpressed in the Arabidopsis *FT* loss-of-function mutant *ft-10*. Arabidopsis *BFT* is thought to be a negative regulator of flowering time, as its overexpression delays flowering time (Chung et al., 2010). Perhaps BrBFT function strictly depends on FT in Arabidopsis, which would have precluded us from observing its function. As *TSF* and *BFT* were proposed to respond to stress in the previous studies (Chung et al., 2010; Riboni et al., 2013), we cannot exclude the possibility that they are involved in abiotic stress–induced flowering.

In addition to the primary function in promoting flowering, BrFTs are crucial in inflorescence organogenesis, as the genetic inactivation of both *BrFT1* and *BrFT2* also impaired floral organ formation in Chinese cabbage (**Figure 4**). A previous study has suggested that loss of Bra.A.FTa (BrFT1) function led to an extreme delay in flowering time but reported no effect on inflorescence architecture (Del Olmo et al., 2019). To explain the discrepancy, we speculate that the FT antagonist TFL1 mainly interacts with FD to form a transcriptional repression complex when both BrFTs are absent, but the presence of *BrFT2* is sufficient to inhibit the formation of the TFL1-FD complex and successfully induce the development of terminal flowers. However, the single mutation of *BrFT1* or *BrFT2* approach to bypass genetic redundancy awaits further clarification. Therefore, it would be interesting to further dissect the possible roles of *BrFT2* in both flowering time and inflorescence organogenesis through molecular and reverse genetic analyses.

What makes a BrFT-like protein function in flowering? FT is translated in leaves and is then transported to the shoot apex where it forms a complex with FD to activate the expression of floral meristem identity genes (Jaeger and Wigge, 2007). Therefore, the interaction of FT with FD is essential for its functional roles. Previous studies have shown that FT interacts with TFL1 through a key amino acid and that changing this amino acid can convert the floral activator FT into a TFL1-like floral repressor, and vice versa (Hanzawa et al., 2005; Hou and Yang, 2009). Moreover, several critical residues in FT can also be mutated to confer a TFL-like activity to FT (Ahn et al., 2006; Ho and Weigel, 2014). Although FD interacts with FT through its C terminus (Ryu et al., 2014), it is still unknown whether certain critical residues in FT are responsible for interacting with FD: the potential binding residues are not conserved with BrFT-like proteins. Our discovery that changing three amino acids can convert the reciprocal interaction of BrFT1, BrFT2, and BrBFT with FD (**Figures 5B, C**) suggests that these three amino acids were required for the interaction with FD and for FT function. It remains to be determined how and why *BrTSF* and *BrBFT* genes evolved into encoding proteins with the divergent amino acids at these positions relative to the close relative Arabidopsis.

## Materials and methods

### Plant materials and growth conditions

Arabidopsis (*Arabidopsis thaliana*) Col-0 and *ft-10* seeds were sown on soil after being stratified for 2 days at 4°C in the dark, placed in a growth room (23°C, long-day conditions; 16 h light/8 h dark or short-day conditions; 8 h light/16 h dark), and grown for 8–9 weeks. The flowering phenotype was assessed based on the number of rosette leaves and days until bolting, which were recorded when the length of the main stem was ≥ 0.5 cm. Phenotyping was performed in three independent biological replicates (with at least 10 plants per replicate).

The early-bolting Chinese cabbage (*Brassica rapa* ssp. *pekinensis*) inbred line ‘20’ was used in this study. Seeds were obtained from NongHyup Seed (Anseong, Gyeonggi-do, Korea). Seeds were sown on sterilized soil and placed in a growth room maintained at 23°C and in long-day conditions (16 h light/8 h dark). After 2 weeks, vernalization was initiated by placing the trays in a cold room at 4 ± 1°C and in a 12-h-light/12-h-dark photoperiod for 35 days. After vernalization, the trays were transferred to a vinyl house and grown for 3 months.

### Plasmid construction and generation of transgenic plants

The full-length regions of *BrFT-like* genes were amplified from ‘20’ Chinese cabbage genomic DNA by PCR with Lamp *Pfu* DNA polymerase (BioFACT, Daejeon, Korea). The PCR products were individually cloned into a modified pCAMBIA1300 vector in which the cauliflower mosaic virus (CaMV) 35S promoter and *NOS* terminator had been cloned into the multiple cloning site. All constructs were verified by sequencing and transformed into Agrobacterium (*Agrobacterium tumefaciens*) strain GV3101. The constructs were transformed into *ft-10* (CS9869; ABRC, Columbus, OH, USA) by the floral dip method (Clough and Bent, 1998). T_1_ seeds were sown onto a half-strength Murashige and Skoog (MS) containing 0.5% agar plate containing 25 mg/L hygromycin for the selection of transgenic seedlings. T_2_ plants showing a 3:1 segregation ratio of hygromycin resistance to sensitivity were selected and allowed to self to collect homozygous T_3_ seeds. The expression of *BrFT*s was confirmed by PCR.

### Bioinformatics analysis

Amino acid sequences of Arabidopsis and *Brassica rapa* PEBPs were obtained from TAIR10 (https://www.arabidopsis.org) and the Brassica database (http://brassicadb.cn), respectively. All proteins were used for constructing a phylogenetic tree and sequence alignment. The phylogenetic tree of PEBP proteins was constructed using Bayesian evolutionary analysis with divergence time analysis in BEAST 2.5 software (version 2.7.1) (Bouckaert et al., 2019). The sequence alignment was analyzed using BioEdit (version 7.2). Rice Hd1a (LOC_Os06g06320) sequence was obtained for sequence alignment using the Rice Genome Annotation Project (http://rice.uga.edu).

Transcriptome deep sequencing (RNA-seq) data were analyzed as previously reported (Jung et al., 2021). Gene expression data for the inbred lines ‘4004’ and ‘50’ were used for the analysis of *BrFT* genes and were represented as a heatmap of the normalized read counts from three biological replicates.

### RNA isolation and PCR analysis

Total RNA was extracted from Arabidopsis rosette leaves of 2-week-old seedlings using the Wizprep™ Plant RNA mini Kit (wizbiosolutions, Gyeonggi-do, Korea). Total RNA was treated with RNase-free DNase I (Thermo Fisher scientific, Waltham, MA, USA) to remove traces of genomic DNA. PrimeScript™ RT Master Mix (TaKaRa, Shiga, Japan) was used for first-strand cDNA synthesis. Subsequently, qPCR was performed on a Bio-Rad CFX real-time PCR system (Bio-Rad, Hercules, CA, USA) using SYBR Prime Q-Mastermix (Genetbio, Daejeon, Korea), according to the manufacturer’s instructions. Relative expression levels were determined by normalizing the expression of each gene of interest against *AtACT2* transcript levels. All PCR determinations were performed from at least three different biological replicates, each with three technical replicates, under the same conditions per experiment.

The relative expression levels of *BrFT-like* genes were confirmed in **Figure 1** using the same conditions as a previous study (Jung et al., 2021). The leaves of the two Chinese cabbage inbred lines ‘4004’ and ‘50’ with different flowering times were collected. Total RNA extraction and first-strand cDNA synthesis were performed as above. All primers used in this study are listed in **Table S3**.

### Bimolecular fluorescence complementation assay

The BiFC assay was conducted as previously described (Walter et al., 2004). The *BrFD* and *AtFD* full-length coding sequences were cloned into pSPYCE-35S, and the full-length coding sequences of *BrFT-like* genes (*BrFT1*, *BrFT2*, *BrTSF*, and *BrBFT*) were cloned from cDNA into pSPYNE-35S. The resulting constructs were introduced in Agrobacterium strain GV3101. Positive Agrobacterium colonies were cultured in YEP medium, pelleted by brief centrifugation, and resuspended to a final optical density of 0.8 in infiltration buffer (10 mM MgCl_2_, 10 mM MES-KOH pH 5.7, and 200 µM acetosyringone). The appropriate pairs of cultures were then co-infiltrated with the P19 silencing suppressor into *N. benthamiana* leaves. After 48 h, YFP fluorescence was observed from the infiltrated leaves using a confocal laser scanning microscope (LSM800; Zeiss, Oberkochen, Germany). The settings for the confocal microscope were as follows: GFP, excitation of 488 nm and emission of 509 nm; RFP, excitation of 553 nm and emission of 573 nm.

### Yeast two-hybrid assay

The full-length coding sequences of *BrFT-like* genes (*BrFT1*, *BrFT2*, *BrTSF*, and *BrBFT*) were individually cloned into vector pGBKT7. The full-length coding sequence of *BrFD* (Bra010504) was cloned into pGADT7. The primers used for cloning are listed in **Table S3**. Each construct harboring one *BrFT-*like gene was co-transformed with the *BrFD* or *AtFD* plasmid into yeast strain AH109, and positive colonies were selected on synthetic defined (SD) medium lacking leucine and tryptophan with dextrose (SD –LT) at 28°C for 7 days. Selected colonies were spotted onto agar plates containing either SD –LT, SD –LTH (SD medium lacking leucine, tryptophan, and histidine), or SD –LTH containing 0.5 mM 3-amino-1,2,4-triazole (3-AT). After plating, the cells were allowed to grow for 7 days. SD –LTH or SD –LTH +3-AT plates were used to test for protein−protein interactions. *BrFD* cloned into the pGBKT7 empty vector was used as negative control, and the *OsCYP18-2-BD* and *OsSKIP-AD* constructs were used as a positive control as previously described (Lee et al., 2015).

### CRISPR/Cas9-mediated mutagenesis of *BrFT1* and *BrFT2* and genetic transformation

Cas-Designer (http://www.rgenome.net/cas-designer/) was used to design a specific single-guide RNA (sgRNA) against the Chinese cabbage *FT* genes (Bra022475 and Bra004117). Thereafter, the selected sgRNA (sgRNA: 5′-AAGCCAAGAGTTGAGAT-3′) targeting both *BrFT* genes was synthesized with a restriction enzyme sequence for cloning into the pHAtC vector (Kim et al., 2016) linearized with the restriction enzyme AarI (CACCTGC (4/8) ^). The resulting vector was transformed into Agrobacterium strain LBA4404 strain and then used for transformation of Chinese cabbage plants.

The Chinese cabbage inbred line ‘20’ from NongHyup Seed in Korea (Anseong) was used for transformation according to a previously published method (Lee et al., 2004). Hypocotyls were incubated for ~1–2 days in darkness before being cut into 0.5- to 1-cm-long segments. Co-culture was performed with the transformed Agrobacterium cultures in the dark for 2 days. After washing, the explants were cultivated on callus induction medium (MS salts with 3% [w/v] sucrose, 5 mg/L benzyl adenine [BA], 1 mg/L naphthaleneacetic acid [NAA], and 300 mg/L cefotaxim) in the dark for 3 days. The induced calli were transferred to shoot induction medium (MS salt with 3% [w/v] sucrose, 10 mg/L BA, 1 mg/L cefotaxime, and 10 mg/L hygromycin). Once shoots developed, the plantlets were cultured on root inducing medium (MS salt with 3% [w/v] sucrose, 0.1 mg/L NAA, and 0.1 mg/L gibberellin).

### Statistical analysis

Statistical analyses were performed in GraphPad Prism 8.0.2 (GraphPad Software, San Diego, CA, USA). Phenotypic analysis was performed by analysis of variance (ANOVA), and different lowercase letters indicate significant differences between samples (*P* < 0.05, one-way ANOVA followed by Tukey’s test). For the analysis of RT-qPCR results, statistical significance was based on two-tailed Student’s *t* tests, with differences considered significant at a *P*-value of <0.05 (*P < 0.05, **P < 0.01, and ***P < 0.005), and on ANOVA, with different lowercase letters indicating significant difference between samples (*P* < 0.05, one-way ANOVA followed by Tukey’s test). At least three replicates were performed, and the data are shown as means ± standard error of the mean.

### Accession numbers

Sequence data from this article can be found in the Brassicaceae Database (BRAD) and The Arabidopsis Information Resource (TAIR10) under the following accession numbers: *BrFT1* (Bra022475), *BrFT2* (Bra004117), *BrTSF* (Bra015710), *BrBFT* (Bra010052), *BrFD* (Bra010504), *AtFT* (At1g65480), and *AtFD* (At4g35900). RNA-seq data were previously submitted to the Gene Expression Omnibus (GEO) database (Jung et al., 2021) under GEO accession numbers GSE106444 and GSE139375 and were reanalyzed here.

## Data availability statement

The datasets presented in this study can be found in online repositories. The names of the repository/repositories and accession number(s) can be found in the article/**Supplementary Material**.

## Author contributions

HSC conceived and designed the research. AL performed the biological and genetic experiments. HJP analyzed the previous transcriptome data and performed biological experiments. HP and SHJ helped generate the transgenic plants. MJ advised on CRISPR/Cas9-mediated mutagenesis of Chinese cabbage. Y-SK developed genome-edited transgenic plants. AL, HJP and HSC wrote the manuscript. All authors contributed to the article and approved the submitted version.
