# Supplementary material for: Their C-termini divide Brassica rapa FT-like proteins into FD-interacting and FD-independent proteins that have different effects on the floral transition
Source: Front Plant Sci. 2023 Jan 12;13:1091563. doi: 10.3389/fpls.2022.1091563 (PMC9878124; doi:10.3389/fpls.2022.1091563)
Supplement: Supplementary file 1 [file DataSheet_1.pdf]

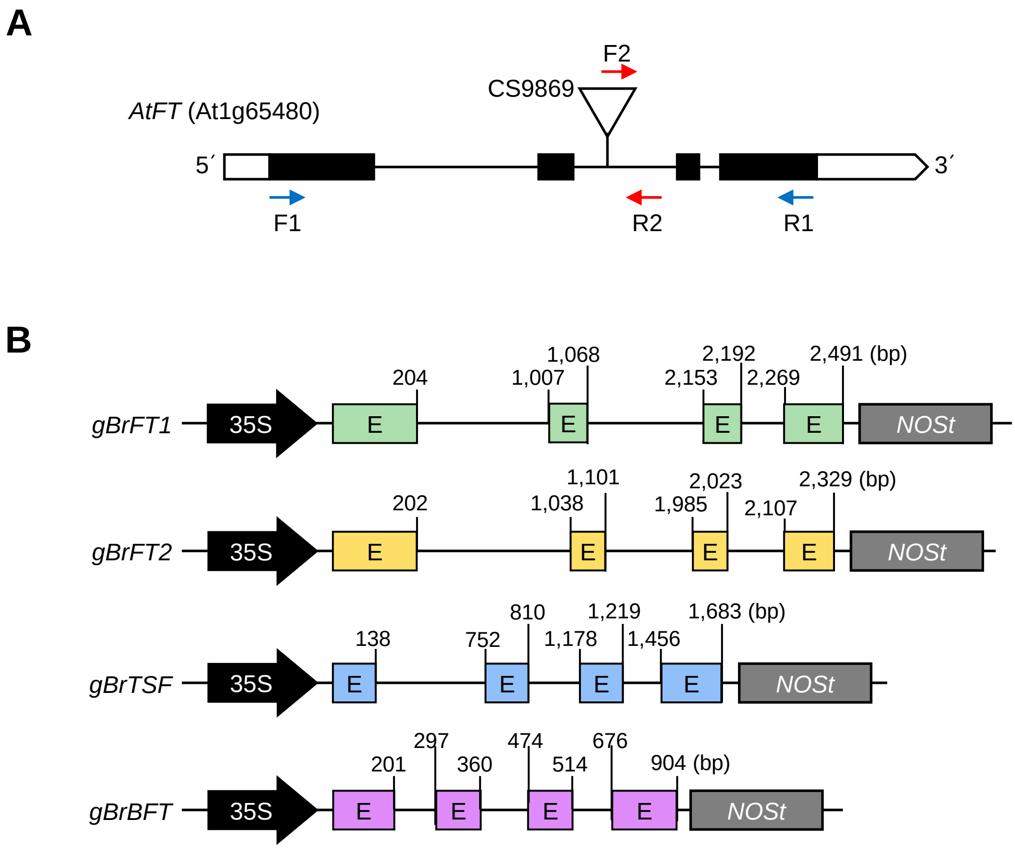

Figure S1. Generation of *BrFT-like* genes overexpressing plant.
(A) Diagram of *AtFT* genomic structure and site of T-DNA insertion (CS9869). Specific primer positions are shown. Exons; black box, intron; black line and untranslated region; white box. **(B)** A schematic illustrating the structure of *BrFT-like* genes cloning vectors (pRG).

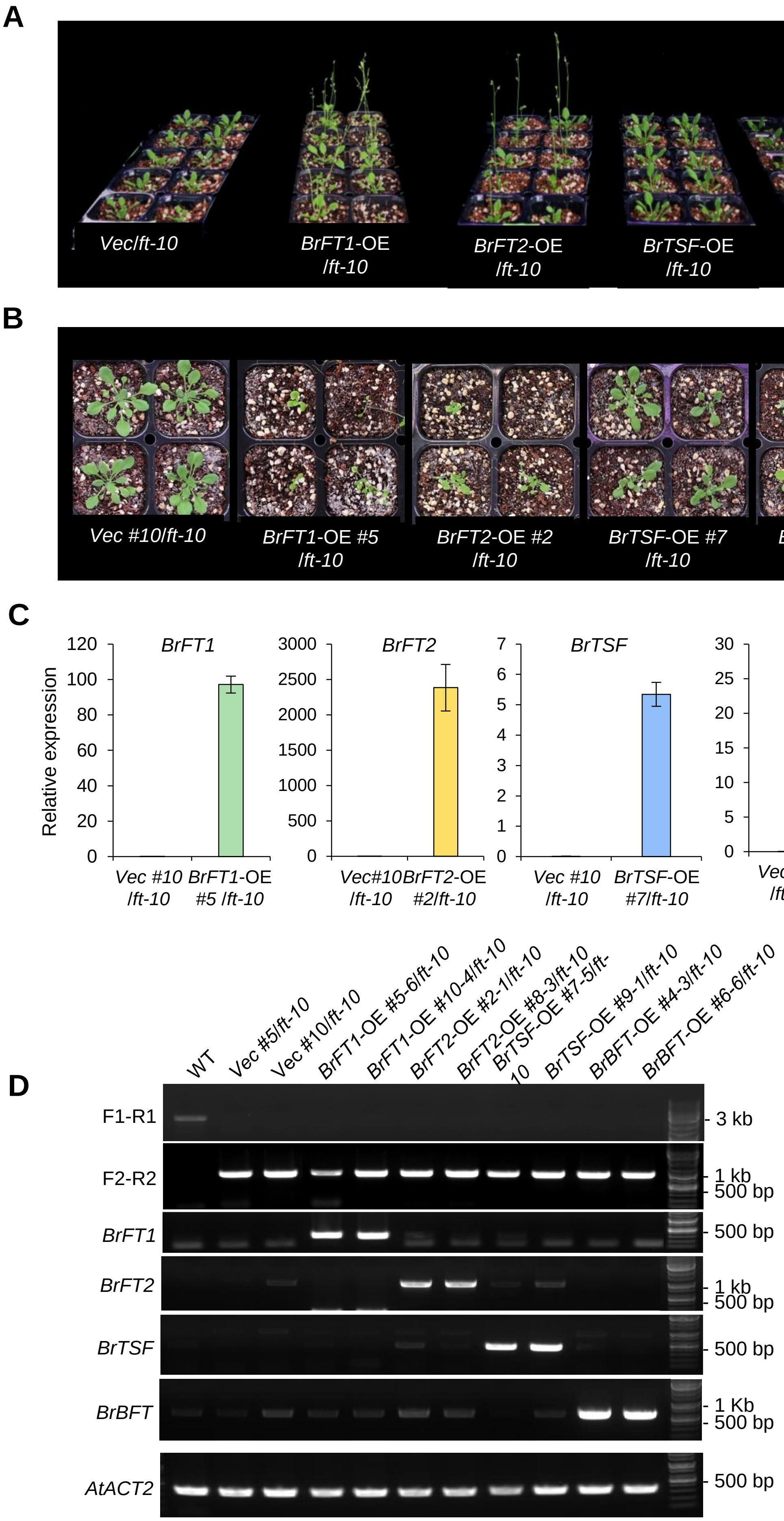
A
Vec/ft-10
BrFT1-OE /ft-10
BrFT2-OE /ft-10
BrTSF-OE /ft-10
BrBFT-OE /ft-10
B
Vec #10/ft-10
BrFT1-OE #5 /ft-10
BrFT2-OE #2 /ft-10
BrTSF-OE #7 /ft-10
BrBFT-OE #4 /ft-10
C
Relative expression
BrFT1
0 20 40 60 80 100 120
Vec #10 /ft-10
BrFT1-OE #5 /ft-10
BrFT2
0 500 1000 1500 2000 2500 3000
Vec#10 /ft-10
BrFT2-OE #2/ft-10
BrTSF
0 1 2 3 4 5 6 7
Vec #10 /ft-10
BrTSF-OE #7/ft-10
BrBFT
0 5 10 15 20 25 30
Vec #10 /ft-10
BrBFT-OE #4/ft-10
D
WT
Vec #5/ft-10
Vec #10/ft-10
BrFT1-OE #5-6/ft-10
BrFT1-OE #10-4/ft-10
BrFT2-OE #2-1/ft-10
BrFT2-OE #8-3/ft-10
BrTSF-OE #7-5/ft-10
BrTSF-OE #9-1/ft-10
BrBFT-OE #4-3/ft-10
BrBFT-OE #6-6/ft-10
F1-R1
F2-R2
BrFT1
BrFT2
BrTSF
BrBFT
AtACT2
3 kb
1 kb
500 bp
500 bp
1 kb
500 bp
500 bp
1 Kb
500 bp
500 bp

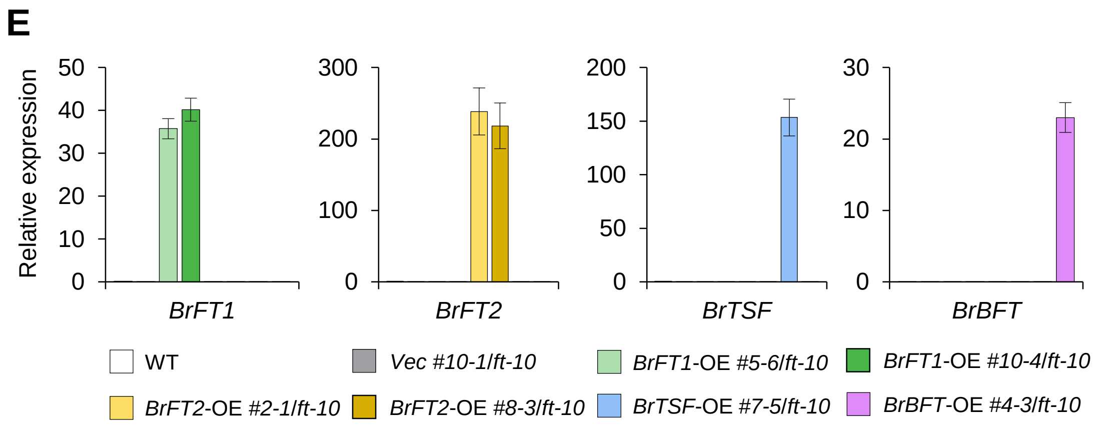

Figure S2. The flowering phenotypes of transgenic Arabidopsis in **(A)** T_1 and **(B)** T_2 generations. Plants were grown for 3 weeks under LD conditions. Bar = 5 cm. **(C)** Confirmation of gene expression in T_2 transgenic plants by RT-qPCR analysis. **(D)** PCR anaylsis using gene specific primers in genomic DNA (gDNA) of T_3 transgenic Arabidopsis. **(E)** RT-qPCR results of *BrFT* genes in WT, Vec #10-1/*ft-10*, *BrFT1*-OE *#5-6/ft-10*, *BrFT1*-OE *#10-4/ft-10, BrFT2*-OE *#2-1/ft-10*, *BrFT2*-OE *#8-3/ft-10, BrTSF*-OE *#7-5/ft-10* and *BrBFT*-OE *#4-3/ft-10* T_3 transgenic Arabidopsis grown under LD conditions for 20 days. Error bars indicate ± SE of three biological replicates.

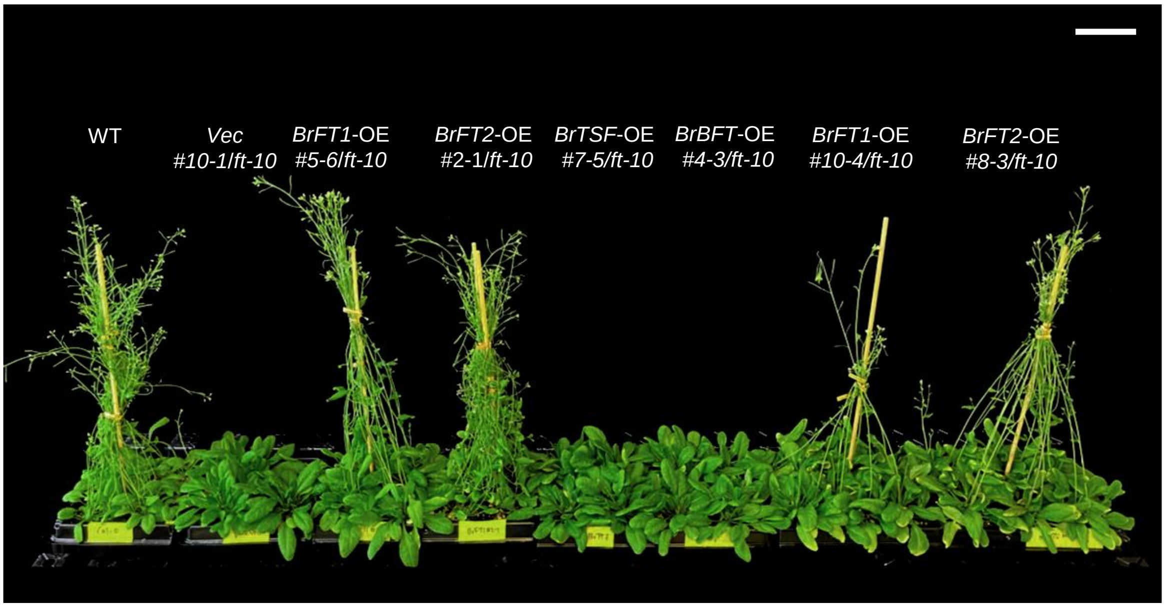

Figure S3. The flowering phenotypes of *BrFT-like* genes transgenic Arabidopsis in T_3 generations. Plants were grown for 6 weeks under LD conditions. Scale bar = 5 cm.

A

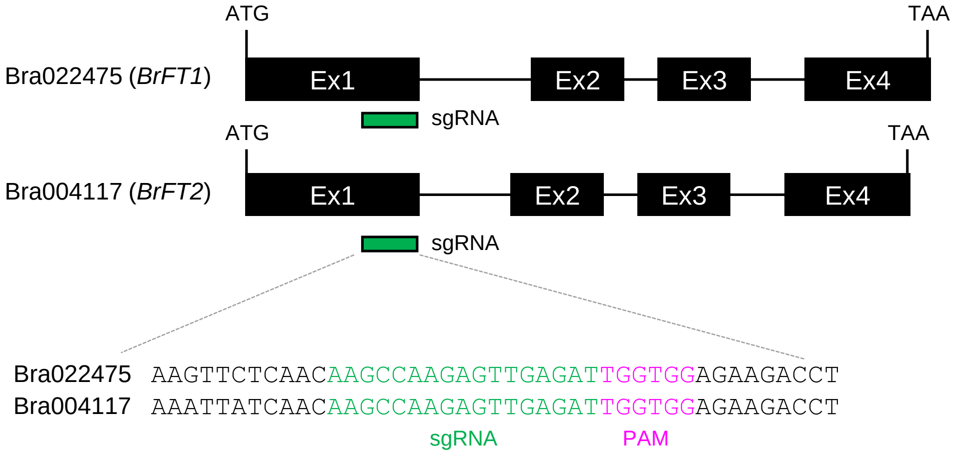

B

Bra022475 (*BrFT1*)

Most frequent sequences	Type	Reads
AAGTTCTCAACAAGCCAAGAGTTGAGATTGGTGGAGAAGACCTAAGGAACTTCT	Wild-type	331
AAGTTCTCAACAAGCCAAGAGTTGAGATTTGGTGGAGAAGACCTAAGGAACTTCT	1 bp insertion(Out of frame)	717,197
	Total	717,528

Bra004117 (*BrFT2*)

Most frequent sequences	Type	Reads
AAATTATCAACAAGCCAAGAGTTGAGATTGGTGGAGAAGACCTAAGGAACTTCT	Wild-type	0
AAATTATCAACAAGCCAAGAGTTGAGAT-------------CT-------TTCT	6 bp deletion(In frame)	270,649
AAATTATCAACAAGCCAAGAGTTGAGATATGGTGGAGAAGACCTAAGGAACTTCT	1 bp insertion(Out of frame)	233,352
	Total	504,001

Figure S4. Genome editing for *BrFT1* and *BrFT2*.
(A) Generation of single-guide RNA (sgRNA) from exon 1 of *BrFT1* and *BrFT2* genomic sequence. **(B)** Sequence analysis of CRISPR/cas9 edited-plants (T_0) by next-generation sequencing (NGS). Sequence colors; Green (sgRNA), Pink (insertion or deletion).

Brad39 (T_0)

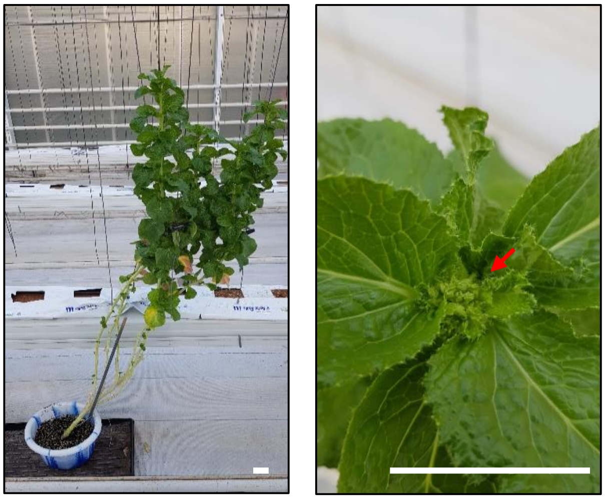

Figure S5. Phenotype of genome-edited plant after 6 months of age. Flower bud development was not normal. Scale bars = 5 cm.

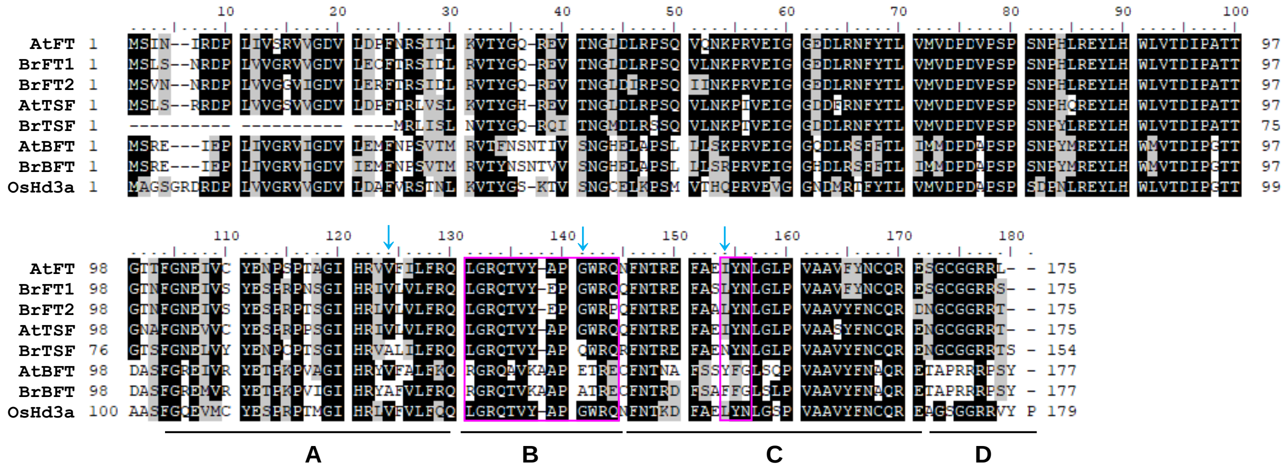

Figure S6. Protein sequence alignment between BrFT-like homologs

Sequence analysis of FT protein homologs from Arabidopsis (AtFT, AtTSF, and AtBFT), rice (OsHd3a) and Chinese cabbage (BrFT1, BrFT2, BrTSF and BrBFT). Two magenta boxes indicate key residues for interaction with bZIP transcription factor FD protein. Alphabet A to D indicate the segment A to B of PEBP domain. The degree of background shading indicates amino acid identity and similarity (black: identity > 60%, gray: similarity > 60%). Protein databases are identified in Materials and Methods.

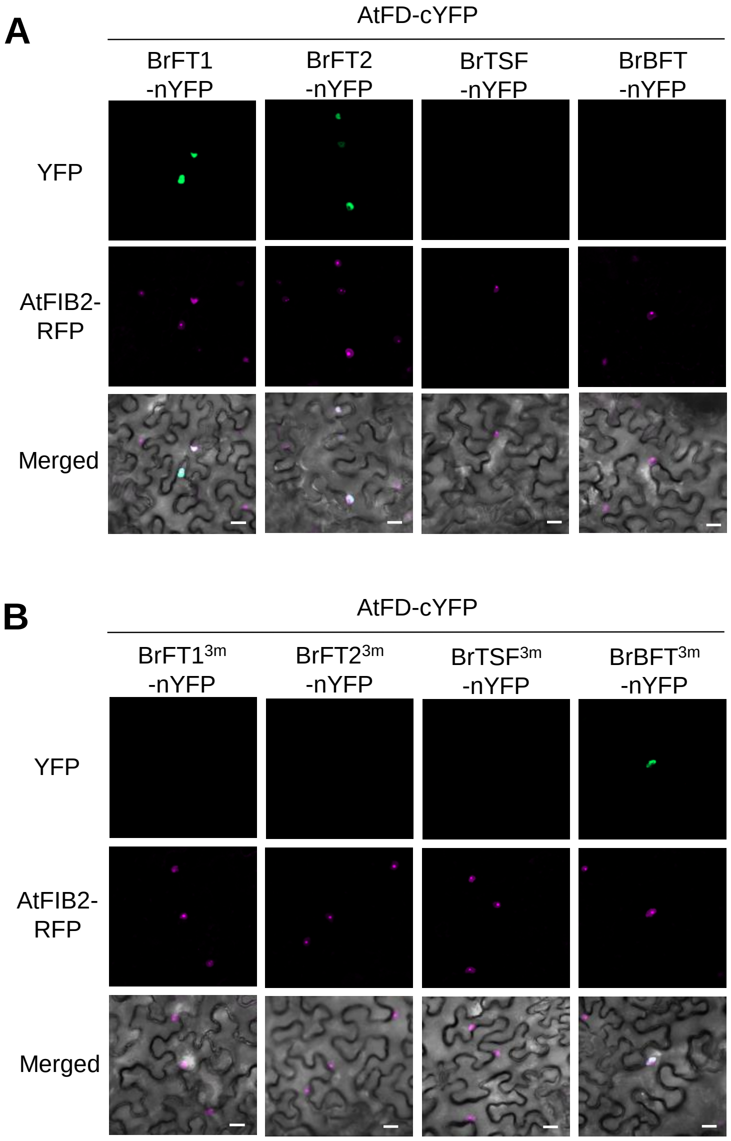

Figure S7 Protein interaction between BrFT-likes and AtFD
(A) BiFC assay of BrFTs fused to N-terminal eYFP (nEYFP) and co-expressed with AtFD fused to C-terminal eYFP (cEYFP) in *N. benthamiana* leaves. Scale bars = 20 μM. **(B)** BiFC assay of BrFT-likes 3-amino acids substitution (BrFT^{3m}s) fused to nEYFP co-expressed with AtFD fused to cEYFP in *N. benthamiana* leaves. Scale bars = 20 μM.

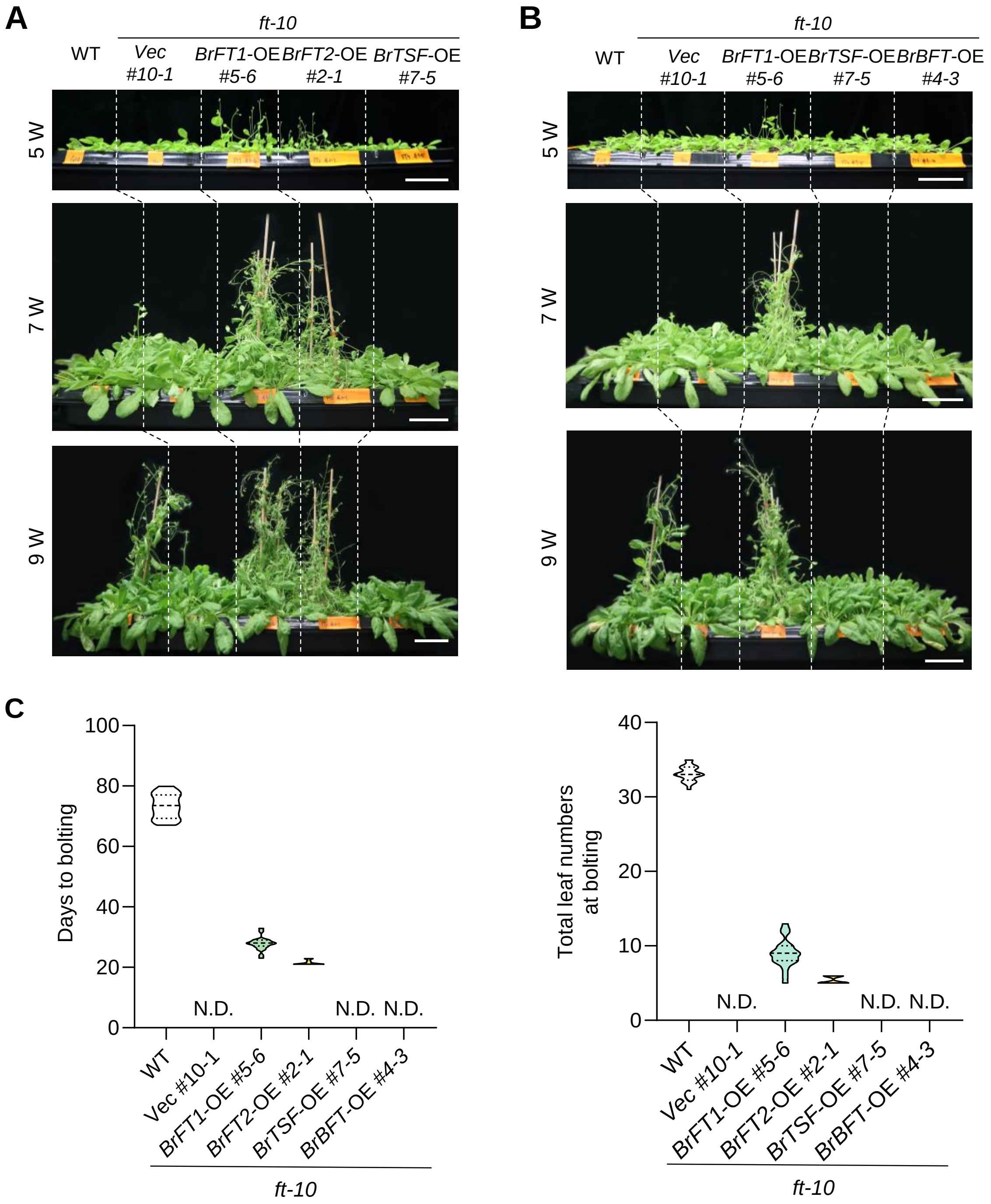

Figure S8. Short day (SD) growth phenotype of *BrFTs* overexpression plants
(A) The ectopic expression lines (*BrFT1*-OE *#5-6/ft-10*, *BrFT2*-OE *#2-1/ft-10* and *BrTSF*-OE *#7-5/ft-10*) and vector control (*Vec #10-1/ft-10*) were compared with the WT. Plant were grown at 23°C under SD conditions for 10 weeks. Scale bar = 5 cm. **(B)** The ectopic expression lines (*BrFT1*-OE *#5-6/ft-10*, *BrTSF*-OE *#7-5/ft-10* and *BrBFT*-OE *#4-3/ft-10*) and *Vec #10-1/ft-10* were compared with the WT. Plant were grown at 23°C under SD conditions for 10 weeks. Scale bar = 5 cm. **(C)** The distribution of flowering phenotypes (Days to bolting and Number of rosette leaves) in T_3 plants with ectopically expressed *BrFTs* were represent in violin plot. Plants of WT, *Vec #10-1/ft-10*, *BrFT1*-OE *#5-6/ft-10* and *BrTSF-OE #7-5/ft-10* were observed more than 20 plants, and plants of *BrFT2-OE #2-1/ft-10* and *BrBFT-OE #4-3/ft-10* were observed more than 10 plants. N.D.; Not detected; Plants were not bolting over 90 days or had more than 35 total leaf number.
